# When fatigue and cognitive impairment persist- a neurological follow-up-study in patients with Post-COVID syndrome

**DOI:** 10.1038/s41598-024-78496-y

**Published:** 2024-11-07

**Authors:** Ann-Katrin Hennemann, Melissa Timmermeister, Nora Drick, Isabell Pink, Karin Weissenborn, Meike Dirks

**Affiliations:** 1 Department of Neurology, Hannover Medical School, Hannover, Germany; 2 Department of Respiratory Medicine and Infectious Diseases, Hannover Medical School, Hannover, Germany

**Keywords:** Post-COVID syndrome, Cognitive impairment, Fatigue, Follow-up, Neurology, Viral infection

## Abstract

Considering the relevance for patients, economics and public health data about the course of the neurological Post-COVID Syndrome (PCS) are urgently needed. In this study 94 PCS patients (73% female, age in median 49 years) were examined in median 9.4 (T1) and for a second time 14 months (T2) after mild to moderate SARS-CoV-2 infection. Mood, sleep quality and health related quality of life (QoL) were evaluated via structured anamnesis and self-report questionnaires; attention, concentration and memory via psychometric tests. 47% of the patients reported an improvement of their symptoms over time, but only 12% full recovery. 4% noticed deterioration and 49% no change. Main disturbances at both time points were fatigue, deficits in concentration and memory. In patients with perceived improvement QoL significantly increased between T1 and T2, although their test performance as well as the fatigue score remained unchanged. In patients with persisting impairment QoL, fatigue scores and psychometric test results did not change significantly. Abnormal psychometric tests were more frequent at both time points in the group without improvement. But, significant fatigue and cognitive impairment persisted for more than 1 year after SARS-CoV-2 infection in both groups.

## Introduction

In case of persistent or newly developed clinical symptoms such as fatigue or cognitive dysfunction more than 3 months after a severe acute respiratory syndrome coronavirus type 2 (SARS-CoV-2) infection and exclusion of other possible causes a diagnosis of a neurological Post-COVID Syndrome (PCS) can be made^1^. Considering the huge number of people who have been infected with SARS-CoV-2, the frequency and clinical course of PCS are of outmost importance, not only for the individuals affected but also for the society since PCS has important socio-economic impact. According to a recent meta-analysis of 68 respectively 43 studies upon the prevalence of fatigue and cognitive impairment more than 3 months after SARS-CoV-2 infection, 32% of the examined individuals reported fatigue and 22% cognitive impairment. Further analysis showed that neither sex nor the severity of COVID-19 in the acute phase had significant impact upon the risk to develop PCS^2^. Of interest there was also no significant difference regarding the prevalence of fatigue and cognitive impairment between those who had been studied less than 6 months or ≥ 6 months after the infection, indicating a risk that PCS might persist in the long-term^2^.

Indeed, PCS has only rarely been studied in long-term follow-up but mostly in cross-sectional studies that address frequency and extent of the different symptoms^3,4^. A French group analyzed the course of 53 symptoms present after SARS-CoV-2 infection in 968 patients over 1 year^5^. The prevalence of symptoms like loss of taste and smell decreased over time, whereas the prevalence of other symptoms – like paresthesia or fatigue - was stable or even increased. A recent analysis of US Veterans Health administration data showed a decrease of various COVID-19 associated symptoms over a time span of 2 years after the infection, but still a significantly higher prevalence of some of them – including fatigue – in those who had been infected compared to subjects who had not^6^. Detailed follow-up data of cognitive function in PCS patients is rare^7^. Recently, Guillen et al^8^. reported a follow-up study in 49 PCS patients that included a detailed neurocognitive assessment. At baseline, which took place about 10 months after the infection (T1), only 25% of the patients achieved normal results in all tests. The number increased after 6 months follow-up to 50% although the test scores only slightly improved, indicating that they had been nearly normal at T1. Fatigue, mood and health-related quality of life remained unchanged.

This study reports a single center longitudinal observation of patients with PCS after mild to moderate COVID-19 disease with assessments in median 9 and 14 months after COVID-19. Patients underwent a structured anamnesis, a neurological examination and a comprehensive psychometric assessment of mood, sleep, health related quality of life (HRQoL), attention, concentration and memory.

## Methods

### Patients

**Fig. 1:**
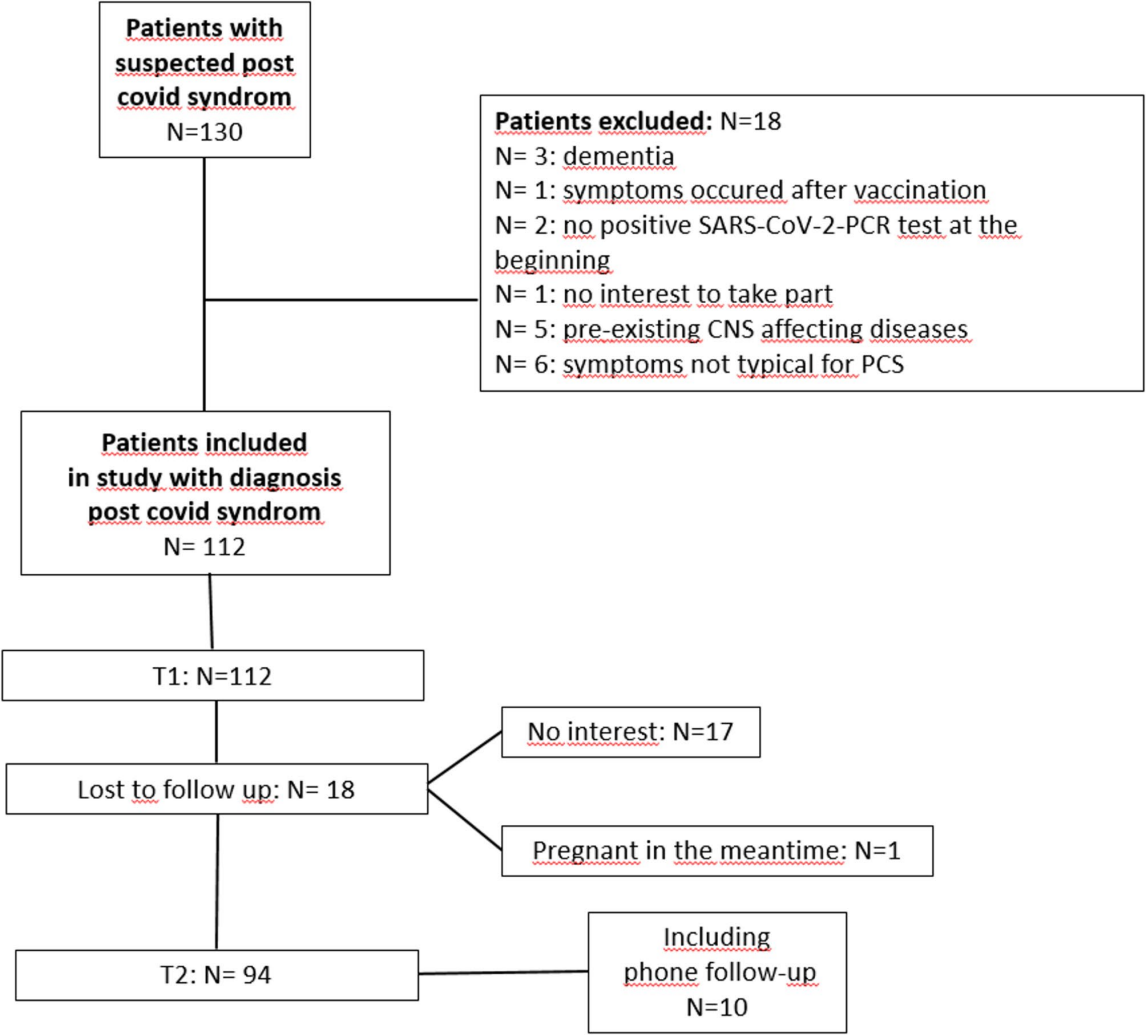
Flow-chart: study inclusion.

One-hundred-and-thirty PCS-patients were assessed between 06/2021 and 06/2023 at the Department of Neurology at Hannover Medical School, Germany. Patients were recruited from referrals by other local departments (e.g. clinic for respiratory medicine and clinic for rehabilitation medicine), as well as from referrals by specialists (neurologists or pulmonologists) or general practitioners. Inclusion criteria were a polymerase chain reaction (PCR) supported COVID-19 diagnosis and persisting symptoms characteristic for PCS for more than 3 months after the infection. Exclusion criteria were age below 18 or above 70 years, medication affecting the central nervous system (CNS), CNS disorders, severe systemic diseases, which might affect brain function, pregnancy or other pre-existing conditions that could contribute to the persisting symptoms. After application of the exclusion criteria 112 patients remained in the study. Ninety-four took part in the follow-up: 84 in person, 10 via phone interview. Eighteen patients were lost to follow-up (Fig. 1).

All patients, except of the 10 who had a phone follow-up, underwent a detailed structured anamnesis and neurological examination as well as a detailed psychometric assessment by an experienced neurologist at baseline and follow-up.

Patients were all tested in the morning between 9 and 12 a.m. All patients completed the psychometric test battery in the same order. The self-assessment questionnaires were given to the patients during their first visit for completion at home.

At both time points the characteristic clinical symptoms of neurological PCS were assessed in detail: olfactory/ taste disorder, fatigue, sleep disorder, concentration and memory deficits, difficulty finding words, headache, myalgia, paraesthesia. Furthermore, self-report questionnaires were completed and a psychometric test battery was performed to objectify the patients’ symptoms. The phone follow-up comprised the structured anamnesis, only.

### Self-report questionnaires

Patients filled in the Fatigue Impact Scale (FIS)^9^, the Hospital Anxiety and Depression Scale (HADS)^10^, Beck’s Depression Inventory (BDI)^11^ and the HRQoL questionnaire SF-36^12^. In addition, daytime sleepiness was assessed with the Epworth Sleepiness Scale (ESS)^13^ and sleep quality with the Pittsburgh Sleep Quality Index (PSQI)^14^.

### Psychometric test battery

To measure global cognitive function the Montreal Cognitive Assessment (MoCA) was used^15^. In addition the Word-Figure-Memory-Test (WFMT)^16^ and the Recurring-Figures-Test (RFT)^17^were applied for the assessment of memory function, and the d2 Test of Attention^18^ and sub-tests from the computer-based attention test battery (TAP) from Zimmermann and Fimm^19^ for the assessment of basal attention and executive function.

The WFMT examines the recognition of words and figures separately, 10 min after they have been presented to the subject. The RFT assesses the short term memory and learning ability for nonverbal material.

The d2 Test of Attention^18^ is a measure for concentration ability, while the subtests from the TAP battery^19^ that were applied in this study test basal responsiveness (“alertness”), cognitive flexibility (“flexibility”) and the ability to focus attention on two tasks simultaneously (“divided attention”).

The single test results were evaluated regarding norm values adjusted for age and education. Test results worse than the 10th percentile of the norm were considered abnormal.

### Statistical analysis

Demographic and clinical data as well as the results of the questionnaires and the psychometric tests was analyzed for normal distribution by Kolmogorov-Smirnov test. Not normally distributed data are shown as median and 25th / 75th percentile, normally distributed as mean (± standard deviation).

The patient group was subdivided into four groups: those who had fully recovered, those who had improved, those who remained unchanged and those who had worsened at follow-up. Pearson Chi-square test was used for testing for group differences regarding sex and infection severity, ANOVA for Body Mass Index (BMI) and the Kruskal Wallis test for the other parameters. Mann Whitney U test was performed as post-hoc test. In a second step the four patient groups were merged into two groups for further detailed analysis: those who had improved since T1 (*N* = 44; fully recovered plus improved patients) and those who had not improved (*N* = 50; patients who remained unchanged or had worsened).

Changes of the psychometric variables between baseline and follow-up were assessed by Wilcoxon signed rank test for repeated measures. McNemar test was used to assess changes for dichotomous variables. Mann-Whitney U-test (for not normally distributed data) and Fishers exact test (for dichotomous variables) were used to test between the follow-up data of the two subgroups.

An univariate and multivariate binary logistic regression analysis was performed with the dichotomized clinical status at follow-up (improved versus not improved) as the outcome variable and age, sex, BMI, education, job (blue collar workers versus white collar workers), timespan from positive PCR to initial assessment (T1) as the exposure variables (unadjusted; model 1 and adjusted; model 2) to obtain odds ratio (OR) with 95% confidence interval (CI). The level of significance was *p* < 0.05. Statistical analysis was performed by using SPSS version 24 and GraphPad Prism (Version 5 for Windows, GraphPad Software, Boston, Massachusetts, USA).

### Ethics approval

The study was approved by the ethics committee of Hannover Medical School (Nr. 1009_BO_S_2021). Following the Declaration of Helsinki written informed consent was obtained from each participant.

## Results

### Baseline characteristics

Of 112 patients seen at baseline 94 took part in the follow-up (73% female, age in median 49 years). The timespan between diagnosis of SARS-CoV-2 infection via PCR and baseline (T1) was in median 9.4 months (25th /75th percentile 7.1/18.1), the timespan between the infection and follow-up 13.9 months (25th /75th percentile 11.2/21.3). Ten patients had been hospitalized for COVID-19 in the acute phase, with *n* = 7 for non-invasive ventilation and *n* = 3 for mechanical ventilation (none with extracorporeal membrane oxygenation). Most of the patients had been infected when the wild-type variant was predominant (see Table 1). A specific variant diagnosis has not been performed.

**Table 1:** Baseline characteristics.

	Study inclusion (T1) (*N* = 112)	Lost to follow-up (*N* = 18)	Entire follow-up-cohort (T1 and T2) (*N* = 94)	Full recovery (*N* = 11) Group 1	Symptomatically improved (*N* = 33) Group 2	Symptomatically unchanged (*N* = 46) Group 3	Symptomatically worsened (*N* = 4) Group 4	*p*-value^a^	Post hoc^b^
Age in years	47 (35/55)	31 (26/52)	49 (39/56)	45 (38/51)	46 (38/56)	51 (41/57)	45 (39/52)	0.584	
Sex female (%)	71.4	61.1	73.4	72.7	81.8	65.2	100	0.238	
Female age in years	47 (36/56)	34 (31/56)	48 (38/56)	44 (35/47)	48 (37/57)	50 (41/56)	45 (39/52)	0.490	
Male age in years	48 (34/55)	28 (23/33)	51 (40/57)	57 (38/57)	46 (38/51)	53 (41/57)	-	0.219	
Timespan pos. PCR to T1 in months	10.3 (7.2/ 17.2)	12.6 (8.4/ 16.8)	9.4 (7.1/ 18.1)	6.7 (5.5/ 12.1)	9.1 (6.3/11.6)	10.9 (7.8 18.9)	19.4 (16/19.6)	**0.007**	1 vs. 4 **0.026** 1 vs. 3 **0.025** 2 vs. 3 **0.048** 2 vs. 4 **0.006**
Timespan pos. PCR to T2 in months			13.9 (11.2/ 21.3)	12.8 (9.5/ 18.7)	12.3 (11.1/ 15.9)	15.1 (11.6/ 23.2)	24.0 (19.5/ 26.3)	**0.015**	
Timespan T1 to T2 in months			3.6 (3.2/4.6)	5.8 (3.3/6.6)	3.5 (3.1/4.2)	3.6 (3.1/4.6)	4.6 (3.4/6.6)	0.097	
Years of education	10 (10/13)	10 (10/13)	10 (10/13)	10 (9/13)	12 (10/13)	10 (10/12)	10 (10/12)	0.187	
Mean BMI (kg/m^2^)	28.35 (± 6.34)	25.65 (± 5.90)	28.86 (± 6.33)	28.56 (± 3.97)	28.8 (± 7.53)	29.4 (± 5.94)	23.9 (± 4.01)	0.434	
Infection severity requiring hospita-lization	10.7% (*N* = 12)	11.2% (*N* = 2)	10.6% (*N* = 10)	9.1% (*N* = 1)	9.1% (*N* = 3)	10.9% (*N* = 5)	25.0% (*N* = 1)	0.575	
Time period SARS-CoV-2 variant**:** Wild type Alpha Delta Omicron	*N* = 49 *N* = 18 *N* = 16 *N* = 29	*N* = 6 *N* = 4 *N* = 4 *N* = 4	*N* = 43 *N* = 14 *N* = 12 *N* = 25	*N* = 2 *N* = 2 *N* = 4 *N* = 3	*N* = 14 *N* = 6 *N* = 3 *N* = 10	*N* = 23 *N* = 6 *N* = 5 *N* = 12	- - - *N* = 4		

Results expressed as mean ± SD for normally distributed data or median and 25th -75th percentiles for not normally distributed data.

^a^ ANOVA p for mean body mass index, Pearson Chi-square test p for sex and infection severity, Kruskal Wallis test p for age, timespan positive PCR to T1 and years of education.

^b^ Mann Whitney U test was performed as post hoc test (significant p-values are displayed).

The virus variants probably responsible for the infection are estimated considering the time point of infection.

Abbreviations: PCR = polymerase chain reaction; BMI = Body Mass Index; SARS-CoV-2 = severe acute respiratory syndrome coronavirus type 2.

Table 1 shows the demographic characteristics of the patients including age, sex, body mass index (BMI), education, hospitalization rate, time from COVID-19 diagnosis to T1 and T2 respectively, and time from T1 to follow-up for the whole baseline cohort (*n* = 112), those lost to follow-up (*n* = 18), the entire follow-up cohort (*n* = 94), the patients who stated full recovery (*n* = 11), the patients who stated an improvement but no full recovery (*n* = 33), the patients who were unchanged (*n* = 46) and those whose symptoms had worsened (*n* = 4).

Patients lost to follow-up were younger, less often female, and had a Post-COVID history at T1 of in median 12.6 months compared to 9.4 months in the follow-up group. Those who reported improvement were in the majority female and had a better education than the rest of the follow-up group. The timespan between COVID-19 diagnosis and T1 was significantly shorter for those who recovered and those who improved compared to those whose clinical symptoms worsened or remained unchanged. The time span between COVID-19 diagnosis and T2 was shorter in those who recovered or improved than in those who remained unchanged or did worse than before. Other significant group differences could not be observed.

### Course of clinical symptoms

#### Clinical symptoms in detail

Considering the entire follow-up cohort the most common symptoms at T1 were deficits in concentration (95.7%), fatigue (90.4%) and memory impairment (78.7%). About 50% of the patients complained about difficulties in finding words (53.2%), insomnia (48.9%) and headache (46.8%). Myalgia and Paresthesia were reported in 23.4% and olfactory/ taste disorders in 18.1%.

In the follow-up an improvement of almost all symptoms was detected but deficits in concentration (84.0%), fatigue (80.9%) and memory impairment (63.8%) persisted to a large extent. Also difficulties in finding words (35.1%), insomnia (42.6%) and headache (34.0%) remained for the most part. Myalgia (23.4%), paresthesia and olfactory/ taste disorders (16.0%) which had been less frequent at baseline compared to fatigue and cognitive dysfunction barely changed in extent.

**Fig. 2:**
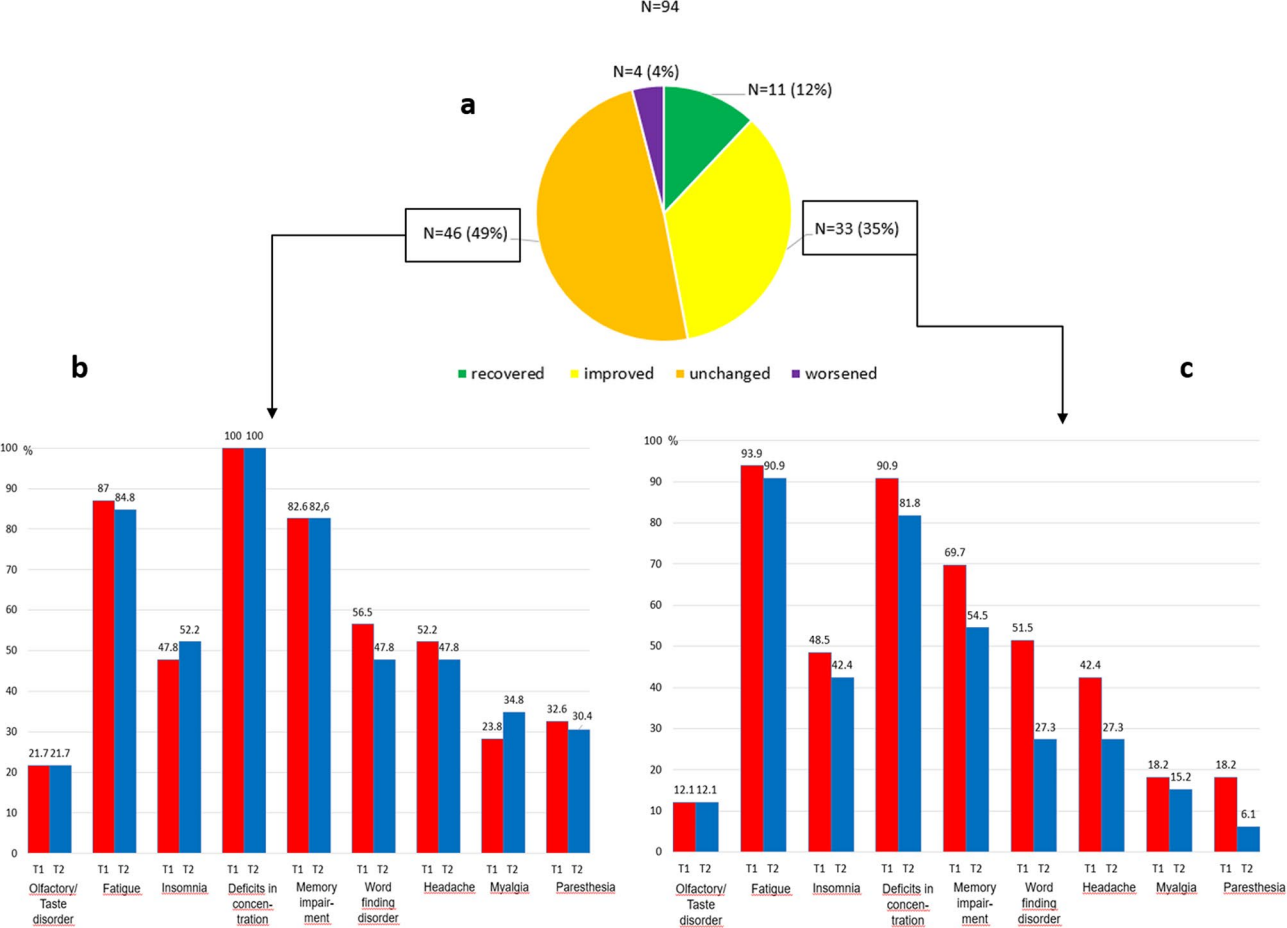
Symptom-oriented assessment at the follow-up (**a**); Course of the symptoms (T1 = red bar, T2 = blue bar) in the subgroup of the symptomatically unchanged (**b**); Course of the symptoms in the subgroup of the symptomatically improved (**c**).

Fourty-four (47%) of the 94 patients stated an improvement of their symptoms at follow-up. Eleven of these were fully recovered. Four reported a worsening and 46 (49%) unchanged symptoms (Fig. 2a).

Looking at the patients with improvement but no full recovery (*n* = 33) difficulties in finding words (T1: 51.5%, T2: 27.3%) and paresthesia (T1: 18.2%, T2: 6.1%) showed the biggest decline, whereas deficits in concentration (T1: 90.9%, T2: 81.8%), fatigue (T1: 93.9%, T2: 90.9%) and memory impairment (T1: 69.7%, T2: 54.5%) barely changed (Fig. 2c).

Patients who reported no improvement complained about the various symptoms recorded as frequently as before (Fig. 2b).

#### Neurocognitive assessment

The neurocognitive assessment at baseline showed only few group differences (Tables 2 and 3). The patient group with full recovery in the follow-up showed lower FIS and depression scores compared to the other groups – despite of similarly low mental and physical SF-36 scores.

**Table 2:** Results of questionnaires at baseline.

	Full recovery Group 1 *N* = 11	Symptomatically improved Group 2 *N* = 32	Symptomatically unchanged Group 3 *N* = 45	Symptomatically worsened Group 4 *N* = 4	*P*-value^a^	Post hoc^b^
FIS	75.0 (56.0–96.0)	96.0 (81.5-112.75)	110.0 (81.5-127.5)	95.5 (56.0-141.8)	**0.011**	1 vs. 3 **0.002**
HADS- D	5.0 (3.0–9.0)	7.0 (4.25–9.75)	9.0 (6.5–12.00)	6.5(3.5-8.0)	**0.043**	1 vs. 3 **0.039** 2 vs. 3 **0.035**
HADS- A	8.0 (4.0–11.0)	7.5 (5.0-10.75)	8.0 (6.0–12.0)	7.0 (4.8–8.5)	0.595	
BDI	11.0 (6.0–14.0)	14.0 (11.3–18.0)	17.0 (11.5–24.5)	16.0 (13.5–27.5)	0.064	
ESS	10.0 (8.0–12.0)	9.5 (7.0–15.0)	11.0 (7.0–14.0)	9.0 (5.3–18.0)	0.913	
PSQI	8.0 (5.0–11.0)	8.5 (7.0–12.0)	8.0 (7.0–11.0)	11.5 (10.0-13.8)	0.217	
SF-36 sum	327.0 (265.5-459.5)	360.5 (254.4-441.2)	319.0 (196.7-361.5)	325.5 (228.1-523.5)	0.145	
SF-36 physical	158.0 (126.0-240.0)	174.0 (127.0-226.5)	126.0 (94.0-170.0)	109.0 (48.3–277.0)	**0.023**	1 vs. 3 **0.048** 2 vs. 3 **0.005**
SF-36 mental	144.5 (100.0-287.5)	197.0 (118.0-243.4)	158.3 (88.8-219.8)	215.3 (116.9-310.7)	0.394	

Results expressed as median and 25th -75th percentiles.

^a^ Kruskal Wallis test p was used for group differences.

^b^ Mann Whitney U test was performed as post hoc test.

Abbreviations: FIS = Fatigue Impact Scale; HADS D = Hospital Anxiety Scale Depression; HADS A = Hospital Anxiety Scale Anxiety; BDI = Becks Depression Inventory; ESS = Epworth Sleepiness Scale; PSQI = Pittsburg Sleep Quality Index; SF-36 Short-Form 36 questionnaire;.

**Table 3:** Results of psychometric test battery at baseline.

	Full recovery Group 1 *N* = 11	Symptomatically improved Group 2 *N* = 33	Symptomatically unchanged Group 3 *N* = 46	Symptomatically worsened Group 4 *N* = 4	*P*-value^a^
MoCA	28.0 (25.0–29.0) 3/11	26.0 (25.0-27.5) 14/33	27.0 (24.0–29.0) 15/46	26.0 (20.8–29.0) 1/4	0.272
WFMT words z	-0.157 (-1.089-0.532) 0/11	-0.170 (-1.375-0.230) 9/33	-0.558 (-1.273-0.376) 11/46	-0.305 (-1.819-1.347) 2/4	0.875
WFMT figures z	-0.297 (-0.907-0.778) 2/11	-0.372 (-1.111-0.655) 6/33	-0.424 (-1.575-0.686) 14/46	-1.092 (-2.262-1.343) 2/4	0.735
Rec figures geo	0/11	0/33	0/46	1/4	**0.001**
Rec figures nonsense	1/11	1/33	4/46	1/4	0.412
D2 Test of Attention errors (%) ^b^	5.1 (0.9–18.0) 2/8	5.2 (1.8–11.4) 4/31	6.4 (3.2–9.1) 5/41	8.7 (n.a.) 1/3	0.924
D2 Test of Attention items-errors ^b^	363.5 (296.0-408.0) 1/8	375.0 (257.0-436.0) 8/31	309.0 (246.0-375.5) 8/41	367.0 (n.a.) 1/3	0.313
Alertness RT without warning	6/11 (55%)	11/33 (33%)	29/46 (63%)	2/4 (50%)	0.077
Alertness RT with warning	5/11 (45%)	18/33 (55%)	29/46 (63%)	1/4 (25%)	0.390
Phasic Alertness	2/11 (18%)	7/33 (21%)	7/46 (15%)	4/4 (100%)	0.718
Flexibility RT	2/11 (18%)	7/33 (21%)	16/46 (35%)	1/4 (25%)	0.501
Flexibility errors	4/11 (36%)	7/33 (21%)	10/46 (22%)	1/4 (25%)	0.754
Divided attention RT auditory stimuli	8/11 (73%)	19/33 (58%)	30/46 (65%)	1/4 (25%)	0.344
Divided attention RT visual stimuli	2/11 (18%)	5/33 (15%)	11/46 (24%)	1/4 (25%)	0.801
Divided attention errors	1/11 (9%)	7/33 (21%)	11/46 (24%)	2/4 (50%)	0.397
Divided attention misses	1/11 (9%)	11/33 (33%)	21/46 (46%)	2/4 (50%)	0.546

Results expressed as median and 25th -75th percentiles and/ or as number and percentage of abnormal test results.

^a^ Kruskal Wallis test p was used for group differences and Fishers exact test was used for dichotomous variables.

^b^ Group 1 *N* = 8, Group 2 *N* = 31, Group 3 *N* = 41, Group 4 *N* = 3.

Abbreviations: MoCA = Montreal Cognitive Assessment; WFMT = Word-Figure Memory Test; Rec figures geo = Recurring figures geometric subscore; Rec figure nonsense = Recurring figures nonsense subscore; RT = Reaction time.

Eighty-four patients took part in the follow-up neurocognitive assessment, of the remaining ten patients 8 refused another neurocognitive assessment as they felt recovered. Two patients reported worsening of their symptoms and did not manage to show up due to their state of health.

Therefore follow-up results of the neurocognitive assessment and the self-report questionnaires are reported for the patient groups of the symptomatically improved and the symptomatically unchanged (Table 4).

**Table 4:** Follow-Up of the results of questionnaires and psychometric test battery and comparison of the Follow-Up-Data of the results of questionnaires and psychometric test battery.

	Subgroup: symptomatically improved Median (25th -75th percentile) Self-report questionnaires: *N* = 26 Psychometric test battery: *N* = 26			Subgroup: symptomatically unchanged Median (25th -75th percentile) Self-report questionnaires: *N* = 36 Psychometric test battery: *N* = 31			
	Baseline	Follow- up	p-value^a^	Baseline	Follow- up	p-value^a^	p- value ^b^
FIS	96.0 (82.5-114.3)	88.5 (74.0-111.3)	0.139	104.5 (80.3–120.0)	116.0 (88.0-128.0)	0.223	**0.005**
HADS- D	7.0 (4.0-9.3)	7.5 (4.0–10.0)	1.000	9.0 (6.3–12.8)	9.0 (5.0-13.8)	0.705	**0.040**
HADS- A	8.0 (5.0–12.0)	7.0 (4.8–9.3)	0.106	8.5 (6.0–12.0)	9.0 (4.0-12.8)	0.349	0.190
BDI	14.0 (10.5–18.3)	13.0 (8.5–19.5)	0.307	16.5 (11.3–24.8)	18.5 (12.0-24.8)	0.224	0.051
ESS	9.5 (7.0-16.3)	10.0 (7.0-13.5)	0.376	10.0 (7.0–14.0)	11.5 (8.0-16.3)	0.084	0.375
PSQI	8.5 (7.0–12.0)	8.0 (6.0-11.3)	0.372	8.0 (6.0–11.0)	10.0 (8.0–12.0)	0.172	0.134
SF-36 sum	364.0 (271.1-447.3)	400.9 (305.8- 497.3)	**0.008**	320.0 (196.5-369.5)	278.8 (177.1-367.3)	0.174	**0.003**
SF-36 physical	174.0 (127.8-225.5)	184.5 (125.0-225.8)	0.073	126.0 (98.5-174.8)	134.0 (63.3-169.5)	0.308	**0.001**
SF-36 mental	186.8 (116.4-240.1)	223.9 (129.1-263.1)	**0.007**	183.5 (89.8-221.1)	137.8 (84.7-227.8)	0.662	**0.040**
MoCA	26.0 (25.0-27.25) 9/26	27.5 (25.0–29.0) 7/26	**0.012**	27.0 (25.0–28.0) 9/31	27.0 (25.0–29. 0) 8/31	0.496	0.559
WFMT words z	-0.149 (-1.399-0.266) 7/26	0.178 (-0.619-0.597) 4/26	0.107	-0.587 (-1.599-0.388) 8/31	-0.780 (-1.331-0.761) 9/31	0.076	0.936
WFMT figures z	-0.651 (-1.058-0.646) 5/26	-0.506 (-1.391-0.457) 8/26	0.716	-0.626 (-1.566-0.726) 10/31	-0.110 (-1.566-1.241) 10/31	0.050	0.648
Rec figures geo	0/26	0/26	0.9	0/31	1/31	0.9	0.9
Rec figures nonsense	0/26	0/26	0.9	3/31	3/31	0.9	0.9
D2 Test of Attention errors (%)	4.9 (1.8–10.3) 3/26	3.8 (2.0-7.1) 2/26	0.459	6.4 (3.9–9.3) 2/31	4.5 (2.1–7.8) 2/31	0.240	0.559
D2 Test of Attention items-errors	382.0 (267.5–440.0) 6/26	408.5 (317.3-472.5) 2/26	**< 0.001**	315.0 (250.0- 388.0) 6/31	344.00 (273.0-425.0) 7/31	**0.028**	0.062
Alertness RT without warning	9/26	12/26	0.453	20/31	19/31	0.9	0.294
Alertness RT with warning	14/26	13/26	0.9	19/31	18/31	0.9	0.600
Phasic Alertness	6/26	3/26	0.375	4/31	4/31	0.9	0.9
Flexibility RT	5/26	4/26	0.9	10/31	7/31	0.375	0.738
Flexibility errors	4/26	4/26	0.9	7/31	8/31	0.9	0.516
Divided attention RT auditory stimuli	16/26	11/26	0.125	22/31	20/31	0.754	0.115
Divided attention RT visual stimuli	3/26	3/26	0.9	7/31	10/31	0.453	0.111
Divided attention errors	4/26	2/26	0.687	8/31	7/31	0.9	0.160
Divided attention misses	7/26	5/26	0.625	12/31	12/31	0.9	0.150

Results expressed as median and 25th -75th percentiles and/ or as number of abnormal test results.

^a^ P within the group with Wilcoxon test for paired samples. McNemar test was used to assess changes for dichotomous variables (normal/abnormal).

^b^ Mann-Whitney U- test (for not normally distributed data) and Fishers exact test (for dichotomous variables) were used to test between the follow-up data of the two subgroups.

Abbreviations: FIS = Fatigue Impact Scale; HADS D = Hospital Anxiety Scale Depression; HADS A = Hospital Anxiety Scale Anxiety; BDI = Becks Depression Inventory; ESS = Epworth Sleepiness Scale; PSQI = Pittsburg Sleep Quality Index; SF-36 = Short-Form 36 questionnaire; MoCA = Montreal Cognitive Assessment; WFMT = Word-Figure Memory Test; Rec figures geo = Recurring figures geometric subscore; Rec figure nonsense = Recurring figures nonsense subscore; RT = Reaction time.

#### Self-report questionnaires

**Fig. 3:**
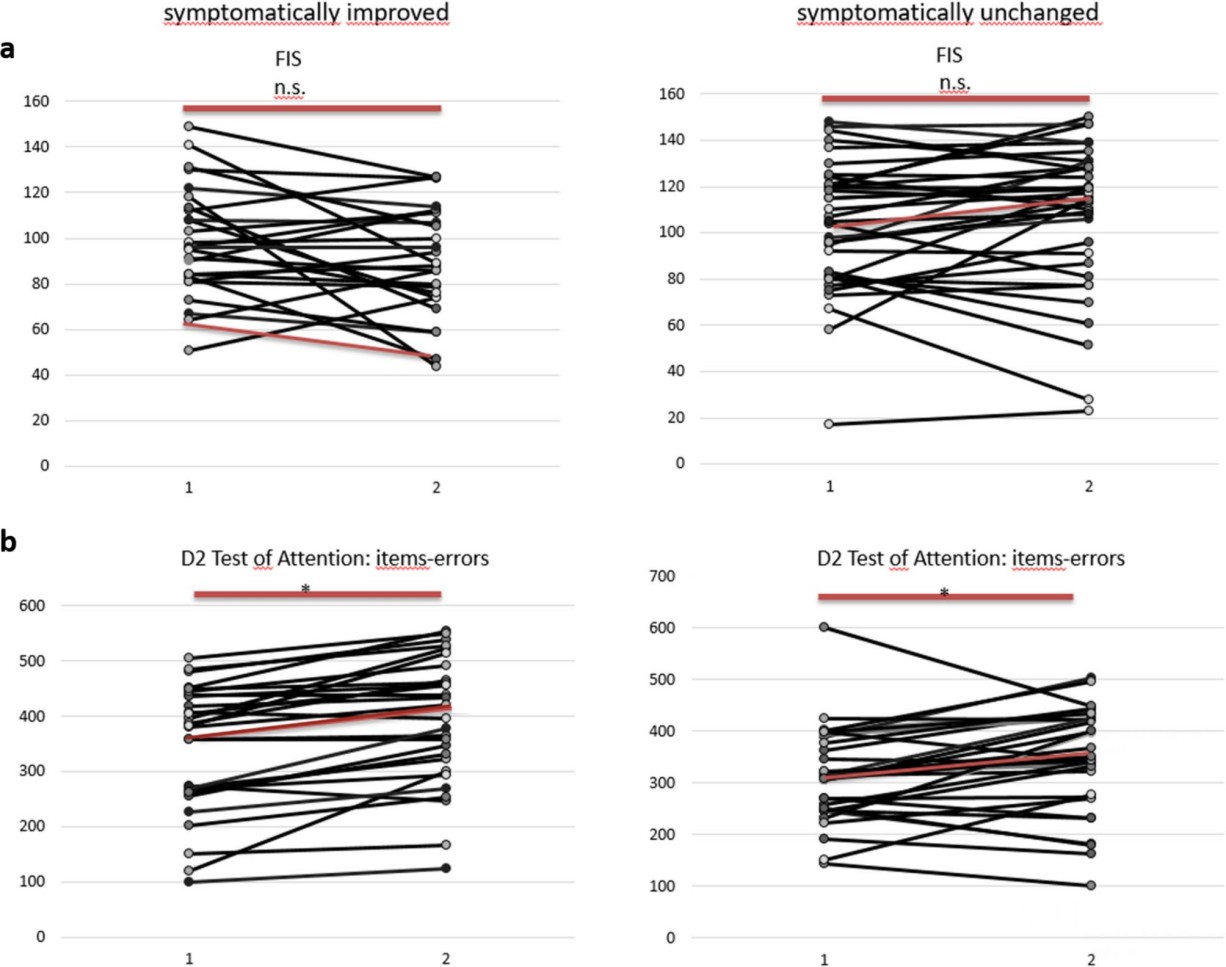
Fatigue Impact Scale (FIS) results in each patient for baseline (1) and follow-up (2). Median results are marked with red dots (**a**). D2 Test of Attention results in each patient for baseline (1) and follow-up (2). Median results are marked with red dots (**b**).

Of 33 patients who reported improvement 26 completed all questionnaires at baseline and follow-up (FIS, HADS, BDI, ESS, PSQI, SF36). At baseline the median FIS, which was stated as sumscore, was high in this group (median 96, cut-off 40; maximum: 160). It improved slightly in the follow-up (median 88.4, *p* = 0.139). The results of the FIS for each individual patient are shown in Fig. 3a. Obviously, the course of the FIS varied between subjects. The BDI score was abnormal at both time points on a similar level (median T1:14, T2:13, normal value ≤ 11), whereas the HADS depression score (median T1: 7, T2: 7.5, normal value ≤ 8) and the HADS anxiety score (median T1:8, T2: 7, normal value ≤ 8) were in the upper normal range. The PSQI was pathological at both time points on a similar level and hints to reduced sleep quality (median T1: 8.5, T2:8, normal value ≤ 5). Daytime sleepiness (ESS) was in the upper normal range at both times indicating that daytime sleepiness is not equal to fatigue (median T1: 9.5, T2:10, normal value ≤ 10). HRQoL (SF-36) was reduced at T1 and T2 but increased in-between (*p* = 0.008) especially regarding the mental score (Table 4). Thus, although the patients reported improvement of their symptoms this was represented only in their HRQoL scores.

Of the 46 symptomatically unchanged patients 36 filled out all questionnaires. The FIS was slightly higher at baseline (median T1: 104.5) than in the former group and even increased in the follow-up (median T2: 116). Individual FIS results are shown in Fig. 3a as well. BDI and HADS depression score were abnormal at both time points (BDI median T1:16.5, T2: 18.5, normal value ≤ 11; HADS depression score median T1:9, T2: 9, normal value ≤ 8), so was the HADS anxiety score (median T1:8.5, T2: 9, normal value ≤ 8). The PSQI was pathological at both times (median T1:8, T2: 10, normal value ≤ 5) and the ESS was in the upper normal range at baseline and follow-up (median T1: 10, T2:11.5, normal value ≤ 10). HRQoL was reduced at baseline and decreased in the follow-up (SF-36 median T1: 320.0, T2: 278.75). None of the observed changes reached the level of significance (Table 4).

Comparing the group of the symptomatically improved and the group of the symptomatically unchanged at follow-up the groups differ significantly regarding FIS, HADS depression score and all SF 36 scores with worse results in the group of the symptomatically unchanged (Table 4).

#### Psychometric test battery

In the group of the symptomatically improved (*n* = 33) the whole test battery was completed twice by 26 patients. Seven patients refused to be tested again. The MoCA result was in median normal at both times in this group and improved significantly from T1 to T2. The results of the WFMT and the RFT were within the normal range at both times and did not change significantly. In the d2 Test of Attention the number of correctly marked items increased significantly (Table 4). The course of the individual test results is shown in Fig. 3b.

The TAP results are shown in Table 4 as well. Part of the patients refused to perform the whole test battery again at T2 or were hindered due to time constraints. As it has been shown before (Table 3) the patients who had reported improvement achieved abnormal results to a noteworthy extent (≥ 50%) in the alertness test and the divided attention test – tests that represent basal responsiveness and the ability to focus attention – at T1. The follow-up assessment does not show any thorough change.

In the group of the symptomatically unchanged (*n* = 46) the complete test battery was performed by 31 patients. Fifteen patients refused to perform the complete test battery because of mental exhaustion and fear to exacerbate fatigue. The MoCA result was within the normal range at both times on a similar level. The WFMT results were within the normal range. The RFT results were only rarely abnormal at both times without significant change. In the d2 Test of Attention the number of items -errors increased (*p* = 0.028) (Table 4); Fig. 3b shows the course of the individual results over time. The results of the TAP battery subtests are nearly unchanged (Table 4).

**Fig. 4:**
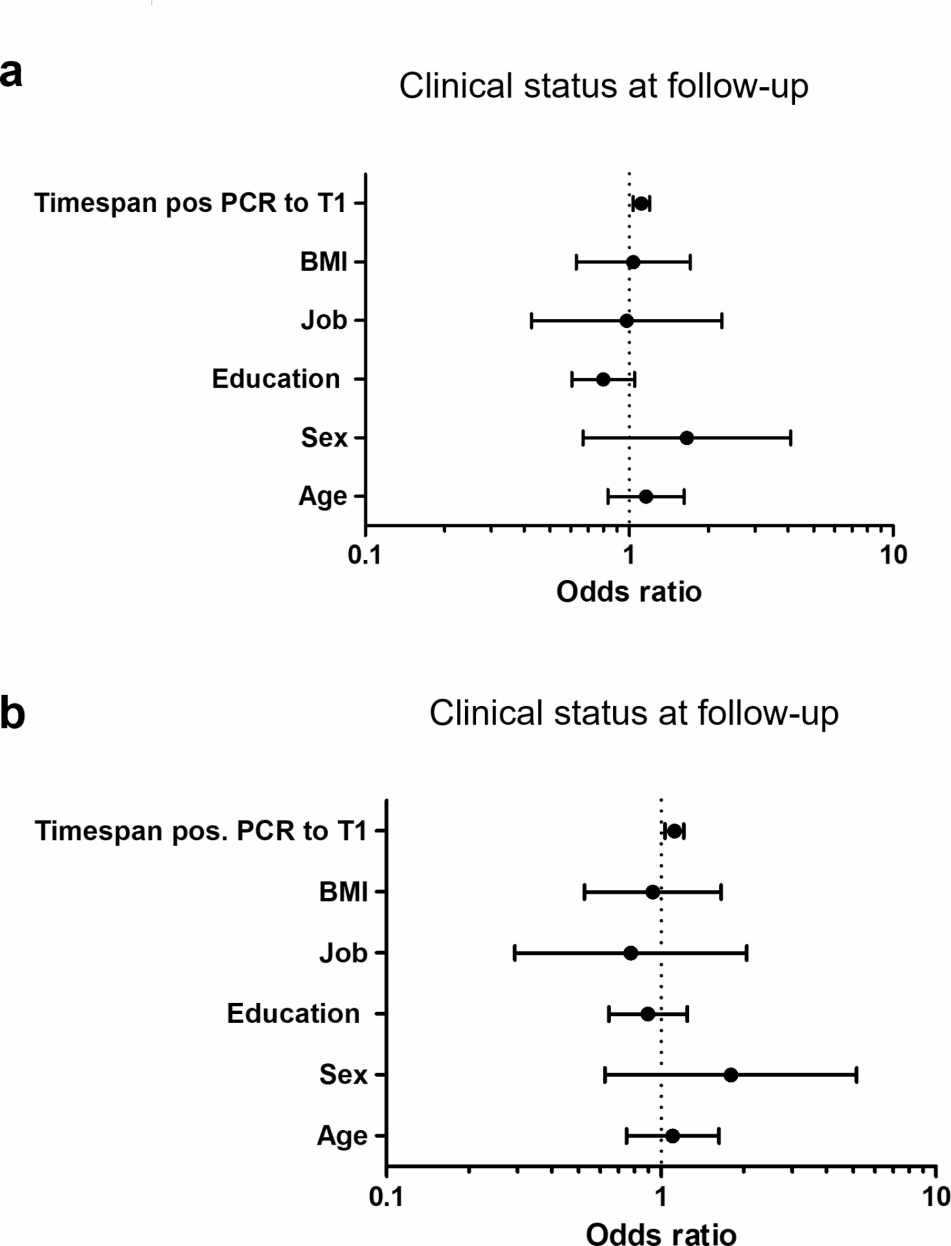
Forest plot for the Odds ratio from logistic regression. The dots represent the Odds ratio (OR). Error bars represent 95% Confidence Interval (CI); log-scale. The dashes lines represent OR = 1. Results unadjusted (**a**) and adjusted (**b**) for age, sex, education, job, BMI, timespan from positive PCR to initial assessment (T1).

Regarding the prediction of clinical outcome at follow-up (improved versus not improved) binary logistic regression analysis was performed with univariate and multivariate models. In both models a longer timespan between COVID-19 diagnosis and initial assessment (T1) predicted no improvement at follow-up (OR = 1.113, 95% CI: 1.036–1.195, *p* = 0.003 and OR = 1.117, 95% CI: 1.033–1.207, *p* = 0.006). Age, sex, BMI, education (in years) and job (blue collar workers versus white collar workers) showed no significant predictive value (Fig. 4).

## Discussion

The Post-COVID-Syndrome has turned out to be a burden for the affected patients as well as a public health challenge with high socioeconomic impact due to persisting and daily life-affecting symptoms^20^. The present study aimed to figure out the persistence of PCS symptoms for more than 12 months. PCS patients who were seen for the first time about 9 months after the COVID-19 diagnosis were re-examined in median 14 months after the infection. Beyond the clinical course neurocognitive function was assessed. The assessment showed a high prevalence of fatigue (80.9%), deficits in concentration (84.0%) and memory impairment (63.8%) in the follow-up examination. Despite of this about 50% of the patients reported an improvement of their symptoms. The other half expressed an unchanged or even worsened status. The vast majority (90.9%) of those patients who reported improvement still complained about disabling fatigue. Symptoms like difficulties in finding words and paresthesia became less frequent, but not fatigue which highlights again the important role of fatigue in PCS.

For the evaluation of the psychometric data the follow-up cohort was divided in two groups - symptomatically improved versus symptomatically unchanged. Patients who reported full recovery or worsening of their symptoms were not evaluated because the groups were very small.

In the group of the symptomatically improved patients the self-report questionnaires reflect the anamnestic reports quite well. In accordance with the patients’ complaints the FIS is very high at both times and improves just to a small extent, while the SF-36 score increases significantly over time as does the health status perceived by the patients. It should be noted, however, that the SF-36 scores are still low compared to age and sex related German norms. This fits to the data of Zhao et al., who also observed low SF-36 scores in a 1-year follow-up of 94 patients suffering from PCS^21^ as well as to the data of Guillen et al^8^., who report even slightly worse SF-36 scores in their 6 months follow-up compared to baseline, which was about 10 months after the infection. The discrepancy between the patients’ health perception represented by increasing especially mental SF-36 scores and the nearly unchanged results of the psychometric testing might be due to only slight improvement in mood and cognitive function that is not captured in the test evaluation as well as improved coping with their situation. In the symptomatically unchanged patients FIS remained on a high level between T1 and T2 in accordance with the patients’ anamnestic health status report.

Fatigue is one of the most common symptoms in Post-COVID Syndrome. In a review Premraj and colleagues^22^ determined the prevalence of neurological and neuropsychiatric symptoms in hospitalized and non-hospitalized PCS patients with an overall prevalence of 37% for fatigue more than 12 weeks after infection. A second review by Sobrino-Relano^23^ revealed similar prevalence for fatigue. Taquet et al^24^. analyzed data from a prospective cohort study in the UK with symptom burden after 6 and 12 months and 2–3 years in patients with clinical diagnosis of Covid-19. Fatigue was one of the most frequently reported symptoms alongside depression, anxiety and subjective cognitive decline. Interestingly fatigue worsened over time.

Although fatigue measured by FIS was high, daytime sleepiness measured by the ESS was not a main symptom. Shanley and colleagues found similar results and claimed that fatigue and daytime sleepiness are distinct symptoms, though related^25^. Daytime sleepiness is considered to result from an impaired arousal mechanism while fatigue is understood as consequence of mental and physical exhaustion^25^. The latter are also represented by the mental and physical SF-36 scores. Here it is remarkable that the mental score in the subjects with no improvement decreased while the physical score remained at a very low level.

Data about neurocognitive function is sparse in the field of PCS. However, most recently, Hampshire and co-workers (2024) published a study of cognitive function in a community based sample of more than 112.000 people^7^. The participants were subdivided according to their SARS-CoV-2 infection status and the duration of symptoms thereafter into: no infection, asymptomatic infection, infection with symptoms for less than 4 weeks, symptoms for 4–12 weeks, symptoms for 12 weeks, symptoms for > 12 weeks. Participants undertook eight computerized online tasks from the Cognitron battery. Participants with symptoms for more than 12 weeks –defined as Post-COVID patients – achieved significantly worse results than those who had not been infected considering the whole cognitive score. Memory, reasoning and executive functions were most affected. This is in line with the study of Arbula et al. who showed in a detailed neuropsychological study of 33 patients versus 27 healthy controls that PCS is associated with significant attention deficits and prospective memory failures^26^. The study was performed about 8.4 months after the COVID-19 diagnosis, thus at a similar time-point as our baseline examination. Hampshire et al. do not mention or discuss the time interval between the patients’ infection and their exam of cognition^7^.

Guillen et al^8^. observed impairment of attention-executive function in 69% of 49 PCS patients and verbal memory impairment in 39%. This is in line with our findings with 13 patients out of 57 (23%) (improved and unchanged group combined) showing abnormal verbal memory function and 32% abnormal figural memory, while 57% (31/57) achieve abnormal results in the attention-executive function tests of the TAP battery. Most former studies use just the MoCA for an estimation of the patients’ general cognitive status^19,20^. Latronico et al. showed in a cohort of 114 patients of COVID-19 associated acute respiratory distress syndrome survivors cognitive impairment according to MoCA in 28% at 3 months after intensive care unit (ICU) discharge^27^. This result is probably biased as nonspecific neurocognitive deficits occur after ICU treatment^28^. Shanley et al. report follow-up MoCA data for 19 patients at 6 months after mild to moderate acute COVID-19. 73.7% showed no change or changed for the better whereas 26.3% got worse compared to baseline^25^. Mendez et al. examined 171 patients by phone call 12 months after acute SARS-CoV-2 infection whereby memory and semantic verbal fluency could be assessed^29^. Similar to the present study cohort 46.8% were considered having persisting neurocognitive impairment.

The assessment of cognitive function may be biased by several factors, such as ICU treatment, concomitant diseases, CNS-affecting medication, mood alterations due to traumatic experiences linked to the pandemic and associated restrictions in everyones’ daily living. To minimize the impact of such limitations the present study focused on patients with mild to moderate COVID-19, while patients with accompanying diseases and medication affecting the CNS were excluded. The HADS scores of the patients were within the normal range or only slightly abnormal, the BDI scores hinted to a minimal or (in the later symptomatically unchanged patients) slight depression. Nevertheless it can be discussed that the cognitive alterations might at least partially be due to concomitant psycho-affective alterations – a consideration that is also made by Arbula et al^26^. Brown et al. even describe a significant association between the severity of depression and cognitive impairment in PCS patients. On the other hand data from Guillen et al^8^. or Schild et al^3^., for example, do not support this hypothesis.

Applying a comprehensive test battery for the assessment of attention and memory deficits, we found abnormal test results in 30–60% of the patients depending on the domain tested. Alertness and divided attention were most frequently affected (Table 3). The patient group without improvement over time showed in addition deficits regarding cognitive flexibility as well as memory disturbances. Of note, the test results did not substantially change in both patient groups during follow-up.

Only the number of correctly marked items in the d2 Test of Attention increased over time, which hints at an improvement of attention ability and concentration in both patient groups, though to a different extent. The significant improvement of their concentration ability might have contributed to the increase of perceived HRQoL in the subgroup of patients who reported improvement of their well-being. However, it must be mentioned that still about half of the patients achieved abnormal results in the alertness test and about 20% in the divided attention test.

Interestingly, also in the group of the symptomatically unchanged the number of processed items - errors in the d2 test increased significantly. The fact that this is not perceived by the patients themselves may be due to their extreme fatigue which might overshadow small neurocognitive improvement. This idea can be underpinned by the fact that this group indeed has higher FIS scores than the group of symptomatically improved patients. Moreover, in contrast to the achieved number of correct items in the d2 Test the results in the TAP battery remained abnormal in a significant portion of the patients, indicating the persistence of attention deficits with impact on executive function. Of note, Bland et al^30^. recently showed a significant impact of perceived stress and fatigue upon the patients’ judgement upon their cognitive abilities. In the same study, they observed that cognitive difficulties following COVID-19 appear to persist in patients with PCS.

In our study, the majority of patients were women (about 73%). In fact, the proportion of women with PCS in population based studies is usually around 2/3 which is consistent with our study^31^. Several studies showed that women suffer more often from PCS and elaborated female sex as a risk factor for developing long-covid^32–34^. In a review Dempsey et al^35^. showed that of 26 studies 8 studies found that females were at higher risk of developing Long-Covid. No study found the male sex to be a risk factor.

Finally, a comment must be made about the patients that were lost to follow-up. They were younger and had a longer time span between infection and first assessment compared to the follow-up group. Reasons for declining a further examination were heterogenous. Some refused to show up again because they felt recovered, others because they feared an escalation of their physical and mental exhaustion.

## Limitations

The refusal of patients, who fully recovered as well as some of those, who are still substantially handicapped by PCS symptoms to take part in the follow-up study impairs the interpretation of the data. Finally, those who are most affected by PCS are not represented. This holds true not only for the present study, but for PCS studies in general.

The number of patients included in the present study is higher than that in other studies on this subject and is obviously sufficient to highlight the clinical characteristics and the clinical course of PCS. Nevertheless a larger cohort could add further important information, such as the impact of the virus variant upon the PCS course, for example^36^.

The data have been elaborated in a single-center observational study. A multi-center approach would probably make the data more generalizable. However, the data achieved in this study are very well in line with others from different European study groups and thus may be considered representative.

## Conclusion

In conclusion, the present data emphasize that symptoms like fatigue, deficits in concentration and memory may persist to a large extent even more than a year after SARS-CoV-2 infection. There is a big need to clarify the pathophysiology behind these symptoms and to develop therapeutic regimens. Data show that the symptoms stated in the case history are represented very well by self-report questionnaires. These seem helpful tools for the long-term assessment of the patients’ status. Further studies are needed to clarify if symptoms indeed persist in PCS patients who are still symptomatic 1 year after COVID-19 or if there still is a chance of improvement. The results of the multivariate binary logistic regression analysis suggest that the chance of symptom improvement decreases with increasing interval from the time point of infection.

## Data Availability

Anonymized data are available on reasonable request.
